# Size‐dependent secretory protein reflux into the cytosol in association with acute endoplasmic reticulum stress

**DOI:** 10.1111/tra.12729

**Published:** 2020-04-13

**Authors:** Patrick Lajoie, Erik L. Snapp

**Affiliations:** ^1^ Department of Anatomy and Cell Biology The University of Western Ontario London Ontario Canada; ^2^ Janelia Research Campus Ashburn Virginia USA

**Keywords:** endoplasmic reticulum, ERAD, fluorescent protein, GFP, photoactivation, signal peptide, intracellular transport, traffic, translocation, UPR

## Abstract

Once secretory proteins have been targeted to the endoplasmic reticulum (ER) lumen, the proteins typically remain partitioned from the cytosol. If the secretory proteins misfold, they can be unfolded and retrotranslocated into the cytosol for destruction by the proteasome by ER‐Associated protein Degradation (ERAD). Here, we report that correctly folded and targeted luminal ER fluorescent protein reporters accumulate in the cytosol during acute misfolded secretory protein stress in yeast. Photoactivation fluorescence microscopy experiments reveal that luminal reporters already localized to the ER relocalize to the cytosol, even in the absence of essential ERAD machinery. We named this process “ER reflux.” Reflux appears to be regulated in a size‐dependent manner for reporters. Interestingly, prior heat shock stress also prevents ER stress‐induced reflux. Together, our findings establish a new ER stress‐regulated pathway for relocalization of small luminal secretory proteins into the cytosol, distinct from the ERAD and preemptive quality control pathways. Importantly, our results highlight the value of fully characterizing the cell biology of reporters and describe a simple modification to maintain luminal ER reporters in the ER during acute ER stress.

## Graphical abstract

Luminal endoplasmic reticulum (ER) environmental reporters must correctly localize to the ER lumen. During misfolded ER protein stress, correctly folded reporter proteins were observed to unexpectedly translocate or reflux from the ER back to the cytoplasm. ER reflux appears to represent a novel retrotranslocation pathway. Reporters could be protected from ER reflux by modestly increasing their size. These findings reinforce the importance of visual confirmation of reporter localization throughout assays and establish a simple solution to maintain reporter localization.

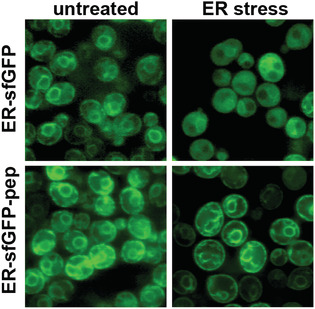

## 1 INTRODUCTION

The standard model of secretory protein localization in eukaryotes holds that proteins are translated in the cytosol and then trafficked to and inserted into the endoplasmic reticulum (ER) membrane or translocated into the ER lumen in co‐ and posttranslational processes.^1^, ^2^ Partitioning of secretory proteins from the cytosol into the ER ensures that secretory proteins fold in the unique ER environment, interact with other partner secretory proteins, and, if appropriate, are secreted out of the ER to other organelles of the secretory pathway or into the extracellular milieu. Secretory proteins that fail to correctly fold can be retrotranslocated from the ER lumen or ER membrane back into the cytosol followed by proteasome mediated destruction in a process termed ER associated degradation (ERAD).^3^, ^4^, ^5^, ^6^, ^7^ Furthermore, some secretory proteins fail to enter the ER because of inefficiencies in the targeting process or sequestration of critical translocation factors.^8^, ^9^, ^10^, ^11^, ^12^

More recently, the Walter group described a condition in which yeast cells impaired in their ability to downregulate the unfolded protein response (UPR) exhibit partial cytosolic localization of a small nonnative ER reporter protein, eroGFP.^13^ The basis of this phenotype is unclear, though the Walter group suggested the cytosolic pool of protein might result from a translocation defect potentially unique to eroGFP, as they reported no altered localization for the resident luminal ER chaperone Kar2 under comparable conditions. As our lab regularly performs live cell imaging studies of fluorescent protein‐tagged reporters and faithful interrogation of the ER requires that the reporter robustly localizes to the ER, we sought to better understand the surprising localization of ER‐targeted eroGFP. Using several reporters with different types of signal peptides, sequences and sizes, we found that the stress‐induced cytosolic pool of accumulation of fluorescent protein (FP) reporters was unrelated to translocation defects. Instead, we uncovered a novel pathway, distinct from ERAD, for the movement of correctly folded soluble ER proteins back to the cytoplasm. A similar concurrent study by the Papa group using the eroGFP reporter drew similar conclusions.^14^ To distinguish this novel ER stress induced mechanism from canonical ERAD pathways, we have termed this phenomenon “ER reflux.”

## 2 RESULTS AND DISCUSSION

### 2.1 Acute ER stress leads to accumulation of ER‐GFP in the cytosol

Our lab has a long‐standing interest in environmental reporters in the ER. An obvious and fundamental requirement for ER reporters and biosensors is that the proteins must actually localize inside the ER lumen to accurately report on the ER luminal environment. Localization to the ER lumen is achieved typically by attaching targeting (signal sequence or SS) and retrieval/retention (‐HDEL) sequences to the reporter. Curiously, we came across a report that described a luminal ER reporter that could be found in the cytosol under ER stress‐related conditions. In the Rubio et al study,^13^ significant cytosolic localization of a redox reporter protein eroGFP (which contained canonical ER‐targeting and retrieval motifs)^15^ was observed in mutant yeast cells with a mutated Ire1 (*ire1(D797N*,*K799N*). Ire1 is the misfolded ER protein sensor and activator of the ER stress pathway, the UPR.^16^ The Ire1 mutant exhibited impaired attenuation of kinase activity following removal of a misfolded secretory protein stress (DTT). Relevant to our study, cytosol‐localized eroGFP was also observed in the absence of application of DTT, which led to the suggestion that these cells had a constitutive translocation defect for secretory proteins. In the following study, we sought to better characterize this phenotype, determine whether it occurred for other ER stresses and reporters, and then attempted to modify ER reporters to improve their ER retention and localization during ER stress.

**FIGURE 1:**
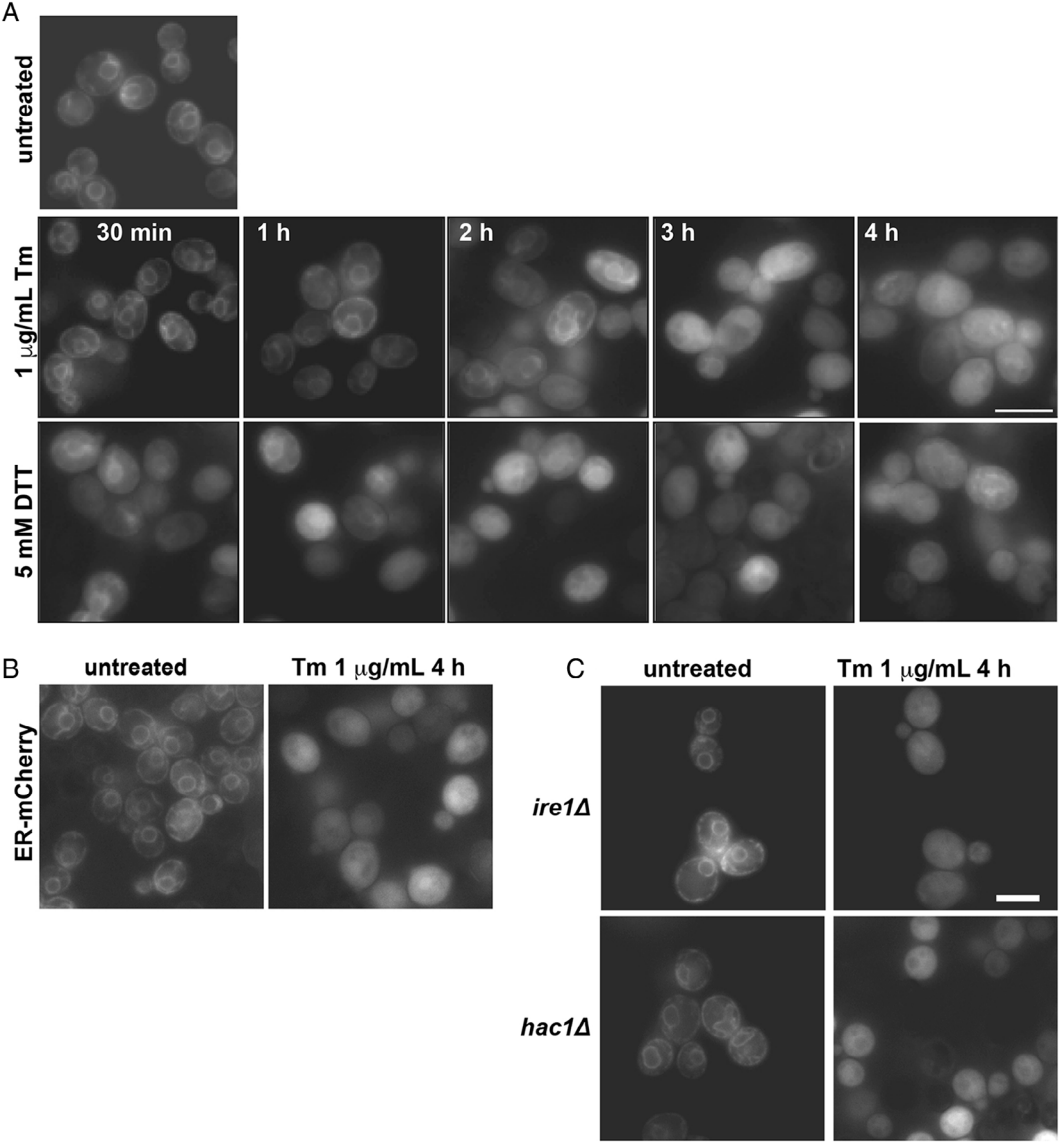
ER‐sfGFP accumulates in the cytosol during acute misfolded secretory protein stress. A, Treatment of strains expressing ER‐sfGFP with secretory protein misfolding agents Tm or DTT lead to localization of ER‐GFP to the cytosol. B, Cytosolic localization also occurs for an ER‐mCherry with Tm treatment. (C) The key UPR effectors are not linked to cytosolic accumulation of ER‐sfGFP. *ire1Δ* and *hac1Δ* cells still exhibit significant cytosolic ER‐sfGFP with Tm treatment. Scale bars = 5 μm

Our first goal was to determine whether ER misfolded protein stresses led to cytosolic localization of ER‐targeted FP reporters. We chose to investigate whether any FP might be susceptible and first focused on a robustly folding FP, superfolder GFP (sfGFP).^17^, ^18^ The sfGFP was targeted to the ER with the Kar2 SS and COOH‐terminal HDEL ER retrieval and localization motif.^19^ We refer to this reporter as ER‐sfGFP. In Figure 1A, we observed ER‐sfGFP localized robustly to the nuclear envelope and peripheral ER in unstressed yeast stably expressing the reporter. Acute treatment with DTT led to accumulation of ER‐sfGFP in the cytosol within 30 minutes of treatment and cytosolic localization became increasingly pronounced with longer treatment times. Similar results were observed with another ER stressor, tunicamycin (Tm), a disruptor of GlcNAc phosphotransferase that impairs N‐linked glycosylation of secretory proteins. Tm treatment took longer to exhibit the cytosolic accumulation and this result is consistent with a delay in impaired N‐linked glycosylation because of the need to first deplete existing stores of dolichol‐bound N‐acetylglucosamine derivatives. Cytosolic accumulation was also observed with an FP with a distinct primary sequence and spectral profile, mCherry^20^ (Figure 1B). Our results suggested that visible accumulation of cytosolic ER‐sfGFP and ER‐mCherry depends on acute ER stress. We next asked whether cytosolic accumulation is a function of misfolded secretory protein build up in the ER and/or the subsequent activation of the UPR. Yeast carrying deletion of genes encoding for either of the two key UPR effectors, *IRE1* or *HAC1*, still accumulate cytosolic ER‐sfGFP with acute misfolded secretory protein stress (Figure 1C). Thus, the cytosolic accumulation of ER‐sfGFP does not rely on a functional UPR.

**FIGURE 2:**
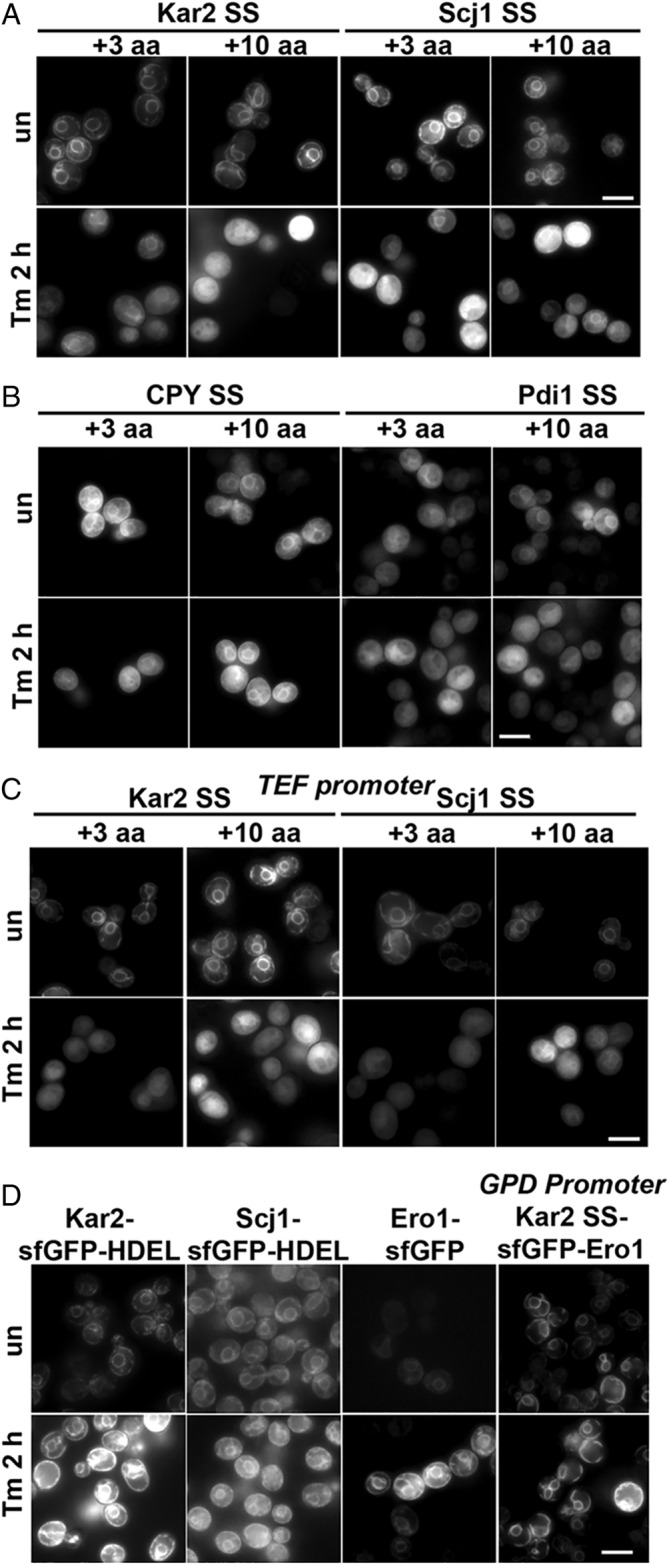
Different SSs and expression levels fail to maintain ER localization of ER‐sfGFP reporters. A, ER‐sfGFP reporters with a variety of different secretory protein SSs accumulate in the cytosol with Tm treatment (5 μg/mL). Addition of 3 or 10 amino acids (+3 or + 10 a.a.) of the mature domain after the SS do not improve ER localization with stress. In some cases, cytosolic localization occurs even in unstressed cells (CPY SS and Pdi1 SS). B, Decreased expression levels with a TEF promoter do not prevent cytosolic localization during Tm stress of ER‐sfGFP reporters with either the Kar2 or Scj1 SS in low copy plasmids. C, Resident ER chaperones fused to sfGFP correctly localize to the ER during acute misfolded protein stress. sfGFP can be placed between the SS and the mature protein domain and Ero1‐sfGFP ER localization remains robust during Tm stress. Scale bars = 5 μm

As a working hypothesis, we speculated that ER stress impairs efficiency of translocation of nascent proteins into the ER. Different signal sequences (SSs) translocate with distinct efficiencies during homeostasis and stress.^8^, ^9^ Therefore, we tested whether the choice of SS affected localization of ER‐sfGFP during stress. We tested six different SSs (see Table 1), including SSs of proteins that translocate co‐translationally (Dap2), post‐translationally (CPY, Pdi1) or both (Kar2).^21^ The mechanism of the yeast HSP40/DnaJ Scj1 translocation is unknown. Two different lengths of mature domains of the proteins immediately following the SS were also included (+3 or + 10 a.a.) because Levine et al reported that including a part of the mature domain of the protein increased translocation efficiency of reporters during unstressed conditions.^8^ We found that some SSs, especially Pdi1 and CPY, localized ER‐sfGFP less well to the ER, even under unstressed conditions. However, all the constructs localized to the cytosol after 2 hours of Tm stress (Figure 2A).

**TABLE 1:** *Saccharomyces cerevisiae* protein signal sequences used in this study

Protein	Sequence (bold letters = SS, *italicized normal letters are in the mature domain*)
Kar2	**MFFNRLSAGKLLVPLSVVLYALFVVILPLQNSFHSSNVLVRG** *ADDVENYGTV*
Pdi1	**MKFSAGAVLSWSSLLLASSVFAQQEAVA** *PEDSAVVKLA*
Ero1	**MRLRTAIATLCLTAFTSA** *TSNNSYIATD*
Prc1 (CPY)	**MKAFTSLLCGLGLSTTLAKA** *ISLQRPLGLD*
Scj1	**MIPKLYIHLILSLLLLPLILAQ** *DYYAILEIDK*
Dap2*	**MEGGEEEVERIPDELFDTKKKHLLDKLIRVGIILVLLIWGTVLLL** *KSI*

a The Dap2 SS is an uncleaved signal anchor.

Another consideration was that the ER‐sfGFP constructs were expressed from multicopy plasmids with a constitutive robust promoter. We hypothesized high ER‐sfGFP expression might overwhelm the translocation machinery during acute ER stress. To test this hypothesis, we decreased expression of ER‐sfGFP using a weaker *TEF* promoter.^22^, ^23^ However, Tm still induced cytosolic accumulation of the ER reporter (Figure 2B).

We next considered the possibility that FPs might contain sequence or structure features that uniquely result in cytosolic localization during ER stress. We have had a long‐standing interest in studying resident ER proteins fused to FPs. Therefore, we were concerned whether an attached FP might be sufficient to target luminal ER proteins to the cytoplasm. We previously tagged endogenous full‐length yeast ER‐chaperones with sfGFP^19^ and did not observe significant cytosolic accumulation during acute ER stress. We retested the Kar2‐sfGFP construct, as well as new full‐length resident ER protein fusions for localization. For all three constructs tested, ER localization was robust in unstressed cells (Figure 2C). Following Tm treatment, we observed a substantial increase in fluorescence intensity, consistent with upregulated expression of the endogenous UPR targets Kar2, Scj1 and Ero1.^24^ However, no significant cytosolic localization was apparent with stress (Figure 2C). Therefore, FPs do not appear to contain dominant information that could alter localization of resident ER proteins during stress.

One caveat is that the position of sfGFP in the fusions was at the end of relatively large proteins. Perhaps, the ER Sec61 translocation machinery might interact more robustly with the sequences of native ER proteins, enhancing translocation efficiency during ER stress. In these fusions, sfGFP might not engage the translocation machinery until after hundreds of amino acids already had been translocated and folded. In contrast, the sfGFP in the simple ER‐sfGFP reporter would engage the translocation machinery immediately after the SS. As GFP was originally a cytosolic protein from jellyfish,^25^ we hypothesized that GFP might lack key protein sequences required for ER entry, specifically during stress. To test this hypothesis, we engineered sfGFP between the Kar2 SS and the mature domain of the robustly ER‐localized resident ER protein Ero1. During Tm treatment, ER localization of the engineered construct was at least as robust as observed with the COOH‐tagged endogenous Ero1‐sfGFP (Figure 2C). Therefore, the presence of sfGFP on a resident ER protein or immediately after the SS did not, in itself impact cytosolic localization during stress. Taken together, two different ER‐targeted FPs fail to correctly localize to the ER during acute stress, while tagged resident ER proteins appear to be unaffected in their localization during ER stress.

**FIGURE 3:**
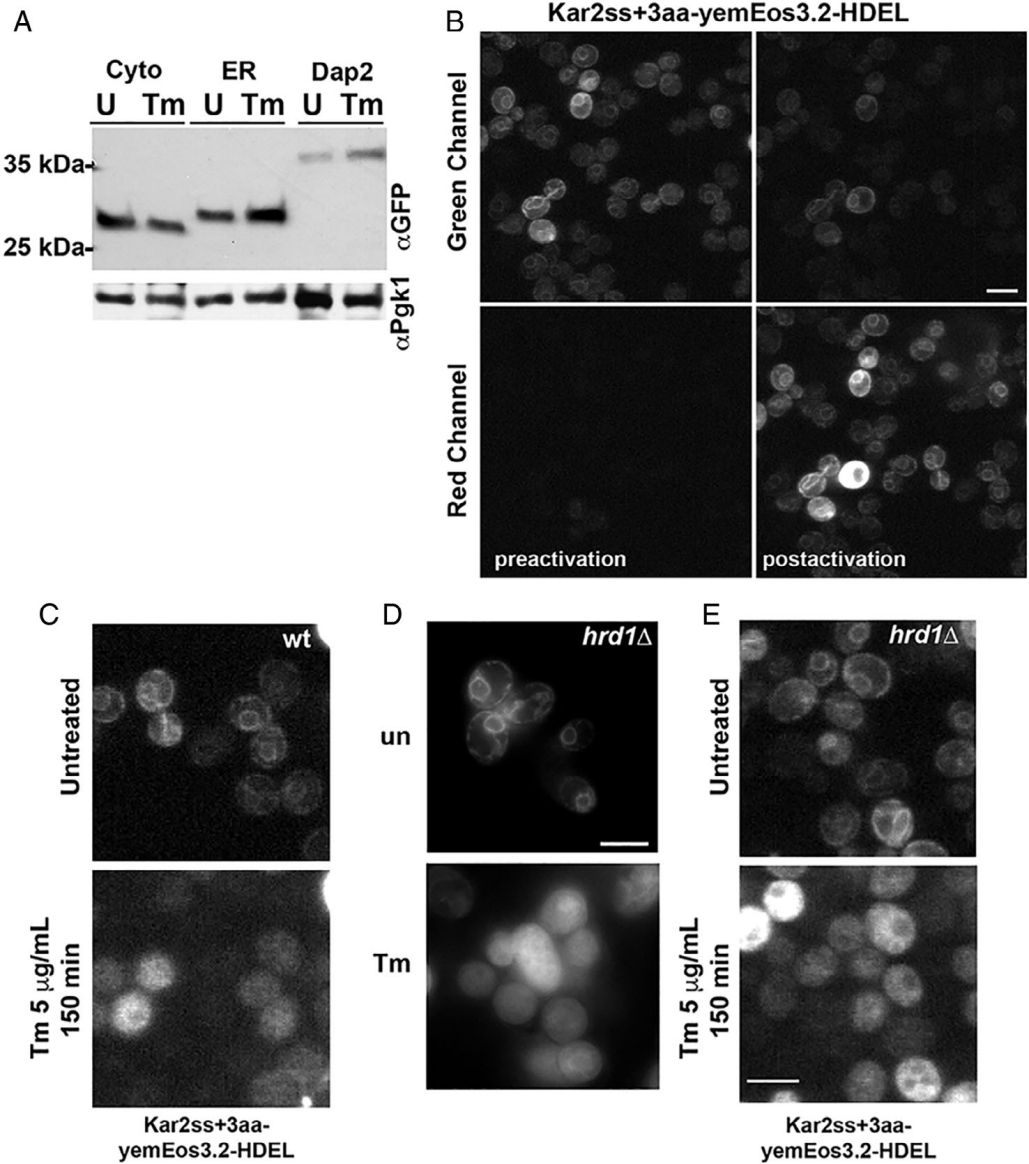
ER reporter that accumulates in the cytosol originated in the ER, but does not appear to rely on ERAD for relocalization. A, Tm treatment (1 μg/mL for 4 hours) does not significantly alter processing of secretory proteins. No size shifts are apparent for ER‐sfGFP relative to the slightly smaller and unprocessed cytosolic GFP or the signal anchored Dap2, suggesting SS cleavage (ER) or lack of cleavage (the uncleaved Dap2 signal anchor) are not significantly impacted during acute stress. Pgk1 serves as a loading control. B, A yeast codon optimized mEos3.2 (yemEos3.2) replaces sfGFP in the ER reporter and can be permanently converted from green to red when cells are briefly photoactivated with a 405 nm laser. C, Reporters in cells were photoactivated to red and then stressed with Tm. Cytosolic accumulation of the ER reporters occurs in both wt and ERAD‐defective cells (*hrd1Δ*). In contrast, inserting a 24 a.a. GS linker into the ER reporter maintains ER localization of the photoconverted red population during Tm stress. D, Cytosolic localization appears to be independent of ERAD‐L. Cells inhibited from ERAD of luminal clients (*hrd1Δ*) still exhibit cytosolic localization of ER‐sfGFP during Tm treatment. E, Similar results are observed for optically highlighted (photoconverted) ER‐yemEos3.2 in *hrd1*Δ cells, which still relocates from the ER lumen to the cytosol following Tm treatment. Scale bars = 5 μm

Following these results, we returned to the initial hypothesis that the cytosolic pool of ER‐sfGFP resulted from a translocation defect. We sought evidence for such a defect. During translocation, many ER SSs are cleaved and this includes the Kar2 SS.^26^ Failure to translocate would likely result in failure to cleave the SS. Therefore, we tested whether the Kar2 SS of ER‐sfGFP was cleaved or uncleaved in stressed cells. Using a 12% tricine gel with sufficient resolution, we compared the sizes of two distinct ER‐sfGFP reporters relative to an unprocessed cytosolic sfGFP‐HDEL in unstressed and stressed cells. In unstressed cells, ER‐sfGFP migrates slightly slower than the unmodified cytosolic sfGFP, which is 8 a.a. shorter (~1 kDa). Thus, the gel resolution can detect at least an 8 a.a. difference in size. One ER‐sfGFP reporter contained an uncleavable the ER‐targeting Dap2 signal anchor (48 a.a. or ~ 5 kDa) that is similar in size to the uncleaved Kar2 SS + 3 (45 a.a.) (Figure 3A). In stressed and unstressed conditions, ER‐sfGFP migrates much faster than the Dap2 SS ER‐sfGFP establishing that the Kar2 SS must be cleaved in both cases, consistent with trafficking to the translocon and cleavage by signal peptidase. The results of the immunoblot argue that Kar2 SS ER‐sfGFP must be translocated into the ER during ER stress for the SS to be cleaved.

This result raised the possibility that ER‐sfGFP might completely translocate into the ER and then be retrotranslocated back into the cytosol, potentially by the ERAD pathway.^3^, ^5^ We tested this hypothesis using two approaches. First, we exploited the power of photoactivatable FPs that can be permanently optically highlighted by laser light to convert the FP from a green to a spectrally distinct red fluorescent species. Photoactivatable FPs can be employed as a visual pulse‐chase reagent to follow protein fate in live cells. Using yeast codon optimized mEos3.2 (yemEos3.2),^27^ we demonstrated that the ER‐targeted FP can be robustly converted from green to red in the yeast ER (Figure 3B). Surprisingly, Tm treatment of cells with photoconverted ER‐mEos3.2 revealed that initially ER‐distributed reporter *relocalized to the cytosol during ER stress* (Figure 3C).

**FIGURE 4:**
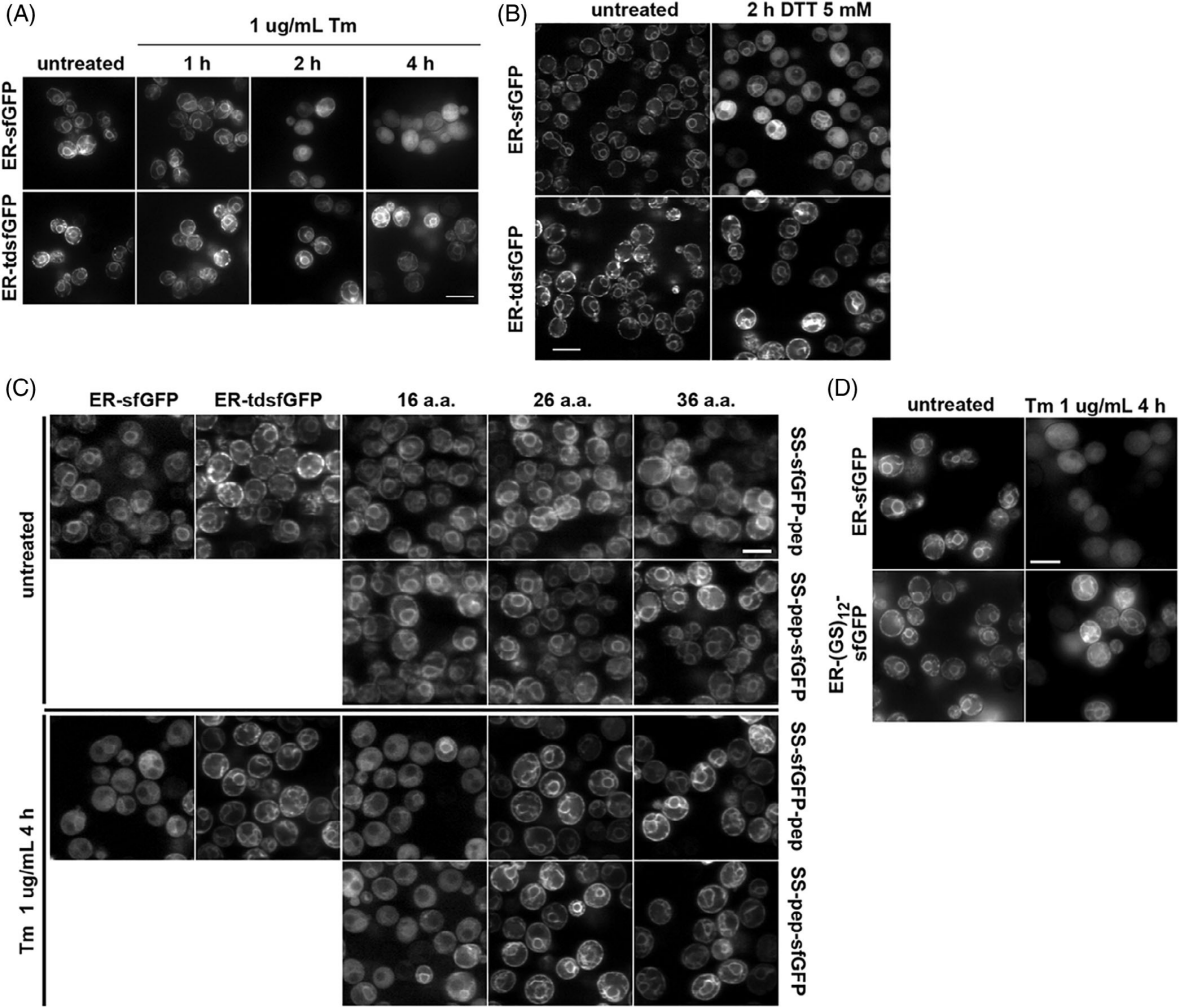
ER localization during misfolded secretory protein stress is improved with increased reporter size. A, Yeast expressing ER‐sfGFP or ER‐tdsfGFP were treated with 1 μg/mL Tm for indicated times and imaged by fluorescence microscopy. B, As in A, but treated with 5 mM DTT. Unlike the other fluorescence micrographs in Figure 4, part B was imaged using confocal microscopy. C, Yeast expressing the indicated constructs were either untreated or treated with Tm for 4 hours. Additions of 26 and 36 a.a. of sfGFP, as a linker, resulted in robust ER localization during Tm treatment. The slightly shorter 16 a.a. linker did not. D, A 24 a.a. non‐GFP linker (Gly‐Ser)_12_ also robustly maintained ER localization of a SS‐pep‐sfGFP reporter during 1 μg/mL Tm after 4 hours. Scale bar = 5 μm

Relocalization or retrotranslocation of an ER protein into the cytoplasm is reminiscent of ERAD, but with two general differences. The first issue was that the reporters did not appear to be incorrectly folded. The standard model of ERAD‐L, the pathway by which luminal proteins are recognized and retrotranslocated to the cytoplasm by the ERAD machinery, generally holds that the luminal target proteins are misfolded and recognized by ER chaperones, such as Kar2 or BiP (in metazoans) or BiP‐associated J protein cofactors.^28^ Given that the FP reporters were fluorescent in the ER argues that the FPs must have first folded into a beta barrel, which is essential for fluorophore formation.^29^ Therefore, to be an ERAD target, the ER FP reporters must be somehow recognized as either unfolded or might somehow be unfolded directly by chaperones. This latter model has precedence for some folded viral proteins and bacterial toxins that ER chaperones have been observed to engage and apparently unfold and help retrotranslocate into the cytoplasm via ERAD.^30^, ^31^, ^32^, ^33^ We will return to this model in Figure 4.

The second issue is that most ERAD‐L clients are destroyed by the proteasome and do not refold in the cytosol.^5^ The retrotranslocation of luminal ER proteins into the cytoplasm is coupled to ubiquitination and subsequent targeting to the proteasome for degradation.^34^ However, bacterial toxins retrotranslocated by ERAD‐L appear to escape destruction in part by a low lysine content, which decreases opportunities for ubiquitination.^30^ FPs contain multiple lysines (20 out of 239 residues in sfGFP, for example) and are susceptible to proteasome degradation when FPs are fused to misfolded proteins.^35^ Taken together, either FPs are retrotranslocated to the cytoplasm by an ERAD mechanism similar to those utilized by viruses and bacterial toxins and/or ERAD is not the retrotranslocation mechanism.

The ERAD‐L pathway requires the membrane protein Hrd1, which is hypothesized to form a channel that can transport proteins from the ER lumen to the cytosol.^34^ Deletion of *hrd1* disrupts ERAD‐L,^36^, ^37^ but did not prevent relocalization of ER‐sfGFP (Figure 3D) or photoactivated ER‐yemEos3.2 (Figure 3E) during Tm treatment. Together, these experiments argue that ER‐sfGFP enters the ER, undergoes SS cleavage and correct folding, and then is retrotranslocated by a mechanism that appears to be distinct from ERAD‐L. Furthermore, these data are in agreement with Igbaria et al that showed deletion of *HRD1*, *DOA10* (which is required for ERAD‐M for membrane proteins), as well as the resulting double mutant do not impair movement into the cytoplasm of ER‐localized GFP reporters during stress.^14^ Thus, the phenomenon appears to be distinct from previously described forms of ERAD. To highlight this difference, we and the lab of Feroz Papa have termed the ERAD‐independent phenomenon “ER reflux.”^14^

### 2.2 A link between ER reflux and heat shock

We turned our attention to whether ER reflux can be stimulated by other acute misfolded protein stresses in cells. We examined whether heat shock can trigger ER reflux and observed no obvious cytosolic accumulation of ER‐sfGFP even after 4 hours of incubating cells at a stressful 40°C (Figure S1A). We also tested whether heat shock might exacerbate or accelerate ER reflux and, surprisingly, found the opposite. Cells treated grown at 40°C and then stressed with Tm exhibited substantial protection against Tm‐induced ER reflux (Figure S1A).^38^ The heat shock response upregulates expression of 165 genes including hlj1, sse1,^39^ which Igbaria et al have implicated in ER reflux.^14^ Interestingly, activation of the heat shock response can alleviate ER stress,^38^, ^40^ suggesting that preventive heat exposure can adapt and enhance the ER folding environment, eliminating a stimulus for reflux.

### 2.3 Engineering an ER reporter resistant to ER reflux

Our original motivation for undertaking this study was to enable interrogation of the ER environment during a variety of conditions including acute ER stress. Therefore, we sought to determine how ER reflux might be circumvented to maintain localization of reporters inside the ER. We looked for a clue in the results in Figure 2 with the various reporters and the ability of tagged endogenous ER proteins to remain in the ER during acute stress. We noted that fusions to full‐length reporters remained ER localized, which suggested the ER proteins might contain retention information and/or that retention might be because of size. All the fusions were significantly larger molecules than GFP alone, ~5 nm.^41^ We tested the size hypothesis by doubling the size of our reporter by making a tandem dimer (td) FP fusion. Consistent with the size‐dependent hypothesis, the new ER‐tdsfGFP exhibited robust ER localization during acute ER stress with Tm or DTT (Figure 4A,B). The larger tdsfGFP also improved ER localization in unstressed cells when the Kar2 SS was replaced with the less robustly ER‐localized Pdi1 SS (Figure S2A,B).

While we were encouraged that we could create a potentially inert, though large, ER FP reporter, we wanted to create a smaller reporter, if possible. In addition, we were curious whether the bulky size of the complete additional GFP β‐barrel was necessary to block ER reflux or whether a shorter truncated peptide would be sufficient to confer resistance to ER reflux. We created a series of ER reporters fused to a second sfGFP truncated at various lengths. In addition, we investigated whether the peptides might be protective on either side of the intact FP. Treatment with Tm led to significant cytosolic accumulation of Kar2 SS ER‐sfGFP fused to the first 16 aa of sfGFP at either the COOH terminus of sfGFP or in between the cleaved SS and fused to the NH_2_ start of the mature domain of Kar2 (Figure 4C). However, we found that addition of a peptide of 26 a.a. or more of the sfGFP sequence was sufficient to prevent ER reflux and maintained ER localization regardless of whether the peptide was placed after the Kar2 SS or at the COOH‐terminus of full length sfGFP, followed by the ER retrieval motif HDEL (Figure 4C). ER localization of the elongated reporter was comparable to ER‐tdsfGFP. Given the lack of a positional requirement for the peptide, we next sought to determine whether the size of the peptide and/or sequence was important for luminal retention. To distinguish between these possibilities, we inserted a sequence encoding a 24mer peptide consisting of (GS)_12_ between the SS and the mature sfGFP. This short hydrophilic peptide also proved sufficient to protect against ER reflux of ER‐sfGFP (Figure 4D). Thus, protein size appears to be more important than sequence for retention in the ER lumen. Finally, we confirmed that preexisting luminal reporters were retained in the ER by adding the SS + 24 a.a. linker to yemEos3.2, expressing it in yeast, photoswitching the reporter from green to red, and then stressing cells expressing the photoconverted protein (Figure S2C). Thus, a short peptide in *cis* can maintain ER‐sfGFP localization during acute misfolded protein stress. This finding should prove extremely helpful for retaining and monitoring biosensors in the ER during acute misfolded protein stress conditions.

**FIGURE 5:**
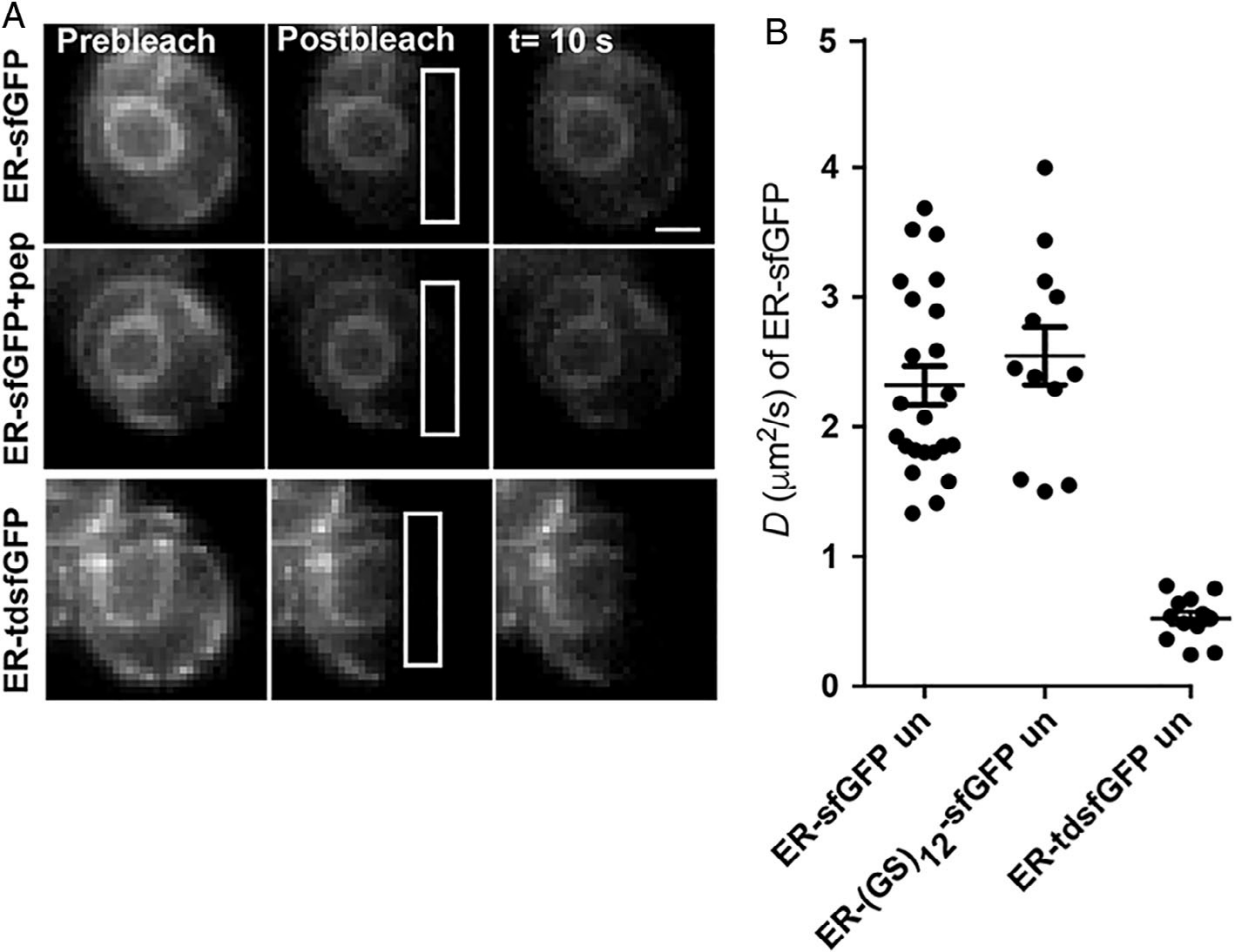
ER‐sfGFP‐pep is a neutral environmental reporter of the ER lumen. A, FRAP time series of yeast expressing ER‐sfGFP‐pep (the +26 a.a. construct from Figure 4) or ER‐tdsfGFP. White ROIs indicate photobleach regions. B, Plot of *D* values for different ER‐sfGFP constructs. Each closed circle marks the *D* value of a single cell. Bars within the dots indicate mean *D* value and SEM. n ≤ 12. Scale bar = 1 μm

One important parameter used for assays of changes in the organization of the ER lumen is viscosity or crowdedness. This parameter can be measured with Fluorescence Recovery after Photobleaching (FRAP) and an inert reporter, such as sfGFP.^18^, ^42^ The need to increase the size of ER‐sfGFP to maintain ER localization made it unclear if reporter mobility would be significantly impacted by the increased size, even in unstressed cells. For, ER‐tdsfGFP, we predicted that linking two sfGFP molecules together (which should result in a relatively large ~10 nm protein) would result in low reporter mobility. We compared the mobilities of ER‐tdsfGFP and ER‐sfGFP‐(GS)_12_ reporters in the ER lumen in FRAP assays. In a homeostatic cell, ER‐sfGFP diffuses at 2.3 μm^2^/s (Figure 5). The Stokes‐Einstein equation predicts that doubling the size of ER‐sfGFP should decrease the diffusion coefficient, *D*, by one half.^43^ However, ER‐tdsfGFP exhibited an extremely low *D* value of 0.5 μm^2^/s or one fifth of ER‐sfGFP. This low value could arise because of an unanticipated interaction with a resident ER protein or more likely because of the relative narrowness of the yeast ER lumen (~10‐76 nm with a mean diameter of 37.9 nm).^44^ Regardless, the low *D* value in homeostatic cells results in a narrow dynamic range for using the tandem dimer reporter to detect changes in ER viscosity during stresses. In contrast, the much shorter reporter, SS‐(GS)_12_‐sfGFP, exhibited indistinguishable diffusion properties relative to ER‐sfGFP (Figure 5B). Thus, addition of a short inert peptide is sufficient to maintain localization and functionality of FP reporters and biosensors in the yeast ER lumen.

### 2.4 ER reflux role and implications for cellular reporters

In this study, we describe a novel phenomenon of ER reflux in which a small soluble ER‐localized protein is expelled from the ER lumen during the acute stressful accumulation of misfolded secretory proteins. Our photoactivation experiments convincingly distinguish this process from some form of failed translocation of nascent secretory proteins. The *hrd1Δ* mutant results strongly suggest ER reflux is distinct from standard models of ERAD. Equally importantly, ER reflux moves a correctly folded protein from the ER to the cytosol and where the protein is also functionally folded. We can draw this conclusion because FPs must form the elaborate β‐barrel structure to form the fluorophore and for fluorescence to occur.^45^ The result with ER‐yemEos3.2 reporter follows a folded green FP in the ER lumen that is converted to a red form and then the converted red form appears in the cytosol. Currently, we cannot rule out the possibility that the reporter unfolds to move from the ER lumen to the cytosol and then refolds.

ER reflux is triggered by acute pharmacologic stresses that induce global misfolding of secretory proteins, but not a cytosolic stress, such as heat shock. Yet, neither an ERAD‐L deletion mutant, *hrd1Δ* (Figure 3D), nor deletion of the key UPR components *IRE1* or *HAC1* appear to cause or prevent ER reflux (Figure 1C), respectively. Together, these observations suggest that yeast possess a novel mechanism for detecting and responding to acute accumulation of misfolded secretory proteins in the ER. However, the release of correctly folded proteins below a molecular size cutoff suggests that ER reflux may not necessarily be a mechanism for clearing the ER lumen of misfolded proteins to restore homeostasis. Most relatively small ER proteins in yeast are integral membrane proteins, which do not appear to be subject to the ER reflux pathway (Feroz Papa and Aeid Igbaria, personal communication).

The role of ER reflux remains unclear. One possibility is that reflux could function to remove otherwise correctly folded and functional small secretory proteins to decrease crowding in the ER lumen. For example, the yeast peptidyl‐prolyl cis‐trans isomerase CPR5, a chaperone, undergoes reflux following Tm treatment.^14^ This result also established that an endogenous substrate can be subject to the ER reflux pathway. Another possibility is that ER reflux could sequester functional proteins from the secretory pathway and block fundamental yeast processes, such as mating/conjugation (ie, Mf(alpha)2 and Mf(alpha)1) (also see Table 2). A curious possibility that we cannot currently rule out is that reporter FPs in yeast could become targets of ER chaperones during conditions of misfolded secretory protein stress. We do not observe binding of FPs by ER chaperones in homeostatic mammalian cells by co‐IP or FRAP or in stressed cells by FRAP analysis.^35^, ^46^, ^47^ We have not tested this possibility by co‐IP in actively stressed cells. The FRAP experiments would be further confounded by the likely impact of chaperone binding of FPs‐namely that the FPs would likely unfold, become dark, and be undetectable by fluorescence microscopy. A major challenge to an FP misfolding model is that both a tandem FP dimer and an FP fused with either a short FP‐derived peptide or a repeating GS peptide of a minimal length are sufficient to prevent ER reflux. It's unclear how a short flexible peptide would protect FP fusions from chaperone binding and unfolding.

**TABLE 2:** Candidate soluble yeast proteins potentially subject to ER reflux

Systematic name	Gene name	Protein length (a.a.)
YGL089C	MF(alpha)2	120
YPL187W	MF(alpha)1	165
YGL258W	VEL1	206
YDR304C	CPR5	225
YOL088C	MPD2	277

As a consequence of our study and the Igbaria et al study,^14^ it is clear that biosensors and FP reporters require validation of functionality and expected localization in cells under the conditions to be explored. Similarly, the impact of different cell environments on biosensor folding and function should not be underestimated.^48^, ^49^, ^50^ In this study, the size of some proteins appears to render the proteins susceptible to relocalization, during misfolded secretory protein stress. Less obvious is the corollary of our findings, tagging of small resident ER proteins with GFP or even a 10 a.a. tag could dramatically alter the ability of proteins to correctly relocalize in response to environmental stimuli. In a similar example, Shao and Hegde described an ER‐targeting and translocation pathway for small (<160 a.a.) secretory proteins.^51^ Small secretory proteins traffic post‐translationally from the cytosol via an interaction with calmodulin to the Sec61 translocon and into the ER lumen. Addition of a GFP or epitope tag would lengthen these proteins and switch trafficking of these proteins from calmodulin dependence over to the co‐translational Signal Recognition Particle‐dependent targeting pathway.

Ultimately, our data argue for caution when using GFP, other FPs or epitope tags to localize or characterize proteins. Raising antibodies against uncharacterized proteins remains an important component of basic cell biology research. Using tagged molecules, an undeniably powerful tool, to characterize proteins requires extreme caution and careful validation that tags do not perturb protein behavior and accurately reflect protein behavior in cells.

## 3 MATERIALS AND METHODS

### 3.1 Drugs

Stock solutions of DTT (1 M in water; Fisher Scientific, Pittsburgh, Pennsylvania) and Tm (5 mg/mL in DMSO; Calbiochem, La Jolla, California) were prepared and used at the indicated concentrations and times indicated.

### 3.2 Strains and cell growth

See Table S1 for all yeast strains used in this study. All strains were derived from BY4741 (*MAT*α *his3Δ0 leu2Δ0 met15Δ0 ura3Δ0*) with transformed plasmids selected by dominant drug markers. The *hrd1Δ* strain was obtained from Dr. Ian Willis (Albert Einstein College of Medicine, New York). The Kar2‐sfGFP‐HDEL strain was made previously.^19^ All yeast strains were grown at 30°C in synthetic complete media supplemented with appropriate amino acids overnight to early log phase (OD600 nm ≈ 0.5) for analysis.

### 3.3 Plasmid constructions

Please see Supplementary Information for plasmid construction information.

### 3.4 Heat shock assay

Yeast cells were grown in the synthetic complete media supplemented with appropriate amino acids at 25°C to early log phase. Cells were either treated with Tm or untreated, and cultured at 30°C or 40°C, followed by fluorescence acquisition at indicated times.

### 3.5 Fluorescence microscopy

After incubation with indicated stresses or stressors, log‐phase cells were placed in 8‐well Lab‐Tek chambers (ThermoFisher Scientific, Waltham, MA) and allowed to settle for 5 minutes before imaging. Cells were imaged in SC complete with appropriate selection components on an Axiovert 200 wide‐field fluorescence microscope (Carl Zeiss MicroImaging, Inc., Thornwood, New York) with a 63X/1.4 NA oil immersion objective lens, a Retiga‐2000 camera (QImaging, Surrey, BC Canada), and 470/40 nm excitation, 525/50 nm emission bandpass filter for GFP, 565/30 nm excitation, or 565/30 nm excitation, 620/60 nm emission bandpass filter for mCherry. Images were acquired with QCapture software. Confocal images were acquired with a Zeiss LSM‐5 LIVE microscope with Duoscan attachment (Carl Zeiss MicroImaging) with a 63X, N.A. 1.4 oil objective and a 489 nm, 100 mW diode laser with a 500 to 550 nm bandpass filter for GFP or a 561 nm diode laser with a 565 nm longpass filter for mCherry. Assessment of FP localization was determined visually. Because of the small size of yeast, scatter, and autofluorescence, cells were scored simply for a nuclear and peripheral ER pattern vs substantial cytosolic and nuclear accumulation, as well as the frequent visibility of the yeast vacuole.

Photobleaching was also performed on the LSM‐5 LIVE. FRAP experiments were performed by photobleaching a region of interest at full laser power of the 489 nm line and monitoring fluorescence loss or recovery over time. No photobleaching of the adjacent cells during the processes was observed. *D* measurements were made using an inhomogeneous diffusion simulation, as described previously.^52^, ^53^ Image analysis was performed with ImageJ (National Institutes of Health, Bethesda, Maryland) and composite figures were prepared using Photoshop CC2018 and Illustrator CC2018 software (Adobe Systems, San Jose, California).

### 3.6 Immunoblots

Early‐log‐phase yeast strains, untreated or treated with Tm, were pelleted and total protein extracted by alkaline lysis.^54^ Lysates were separated on 7.5% or 12% SDS‐PAGE tricine gels, transferred to nitrocellulose membranes and detected with anti‐GFP (from Ramanujan Hegde, MRC Laboratory of Molecular Biology, Cambridge, United Kingdom) and horseradish peroxidase‐labeled anti‐rabbit (Jackson ImmunoResearch Laboratories, West Grove, Pennsylvania).

### 3.7 Statistical analysis

Prism software (GraphPad Software, San Jose, California) was used to compare the different conditions using two‐tailed Student's *t* tests. For higher stringency, differences were not considered significant for *p* values >.01.

**Abbreviations**
a.a.: amino acids
bp: base pairs
*D*: diffusion coefficient
DTT: dithiothreitol
ER: endoplasmic reticulum
ERAD: endoplasmic reticulum associated degradation
FP: fluorescent protein
FRAP: fluorescence recovery after photobleaching
GFP: green fluorescent protein
MFI: mean fluorescence intensity
QC: quality control
sfGFP: superfolder GFP
SS: signal sequence
td: tandem dimer
Tm: tunicamycin
UPR: unfolded protein response
yemEos3.2: yeast codon optimized mEos3.2
yesfGFP: yeast codon optimized superfolder GFP

## Supporting information


**Appendix** **S1**: Supporting information
