# Supplementary material for: Size‐dependent secretory protein reflux into the cytosol in association with acute endoplasmic reticulum stress
Source: Traffic. 2020 Apr 13;21(6):419–29. doi: 10.1111/tra.12729 (PMC7317852; doi:10.1111/tra.12729)
Supplement: Supplementary file 1 — Appendix S1: Supporting information [file TRA-21-419-s001.pdf]

Supplementary material

MATERIALS AND METHODS

Plasmid Constructions

sfGFP-HDEL with the first 135 base pairs of the Kar2 coding sequence, i.e. Kar2 signal sequence (SS) + the first 3 amino acids of the mature domain (Kar2SS+3aa) was described previously.[1] SfGFP-HDEL with the coding sequence of the Kar2 signal sequence plus 10 additional amino acids (Kar2SS+10aa) was made by inserting the first 156 base pairs of the Kar2 coding sequence into the *Bgl*II/*Age*I sites of sfGFP-HDEL-N1 plasmid [2, 3] using the following primers:
Forward, GATCAGATCTCTAAAAATGTTTTTCAACAGAC
Reverse, GATCACCGGTCCAACAGTTCCGTAGTTTTC
The Kar2+10aa-sfGFP-HDEL and Kar2+3aa-sfGFP-HDEL fragments were subsequently amplified using the following primers to introduce yeast Kozak sequences:
Forward, GATCACTAGTCTAAAAATGTTTTTCAACAGAC
Reverse, GATCGGATCCTTACAATTCATCGTG
The resulting fragments were digested and cloned into the *Spe*I/ *BamH*I site of pRS415-GPD (a generous gift from Elizabeth Miller, Columbia University, New York, NY).
sfGFP-HDEL with Kozak sequence and the Cpy signal sequence plus 3 or 10 additional amino acids (CpySS+3aa/CpySS+10aa), Pdi1 signal sequence plus 3, 10 additional amino acids (Pdi1SS+3aa/Pdi1SS+10aa), or Dap2p signal sequence plus 3 additional amino acids (Dap2pSS+3aa) was made by inserting corresponding coding sequences into the *Bgl*II/*Age*I sites of sfGFP-HDEL using the following primers:

CpySS+3aa
Forward, GATCAGATCTCTAAAAATGAAAGCATTCACC
Reverse, GATCACCGGTCCCAATGAGATGGCCTTAG
CpySS+10aa
Forward, GATCAGATCTCTAAAAATGAAAGCATTCACC
Reverse, GATCACCGGTCCATCTAGACCCAACGG
PDI1SS+3aa
Forward, GATCAGATCTCTAAAAATGAAGTTTTCTGCTG
Reverse, GATCACCGGTCCCTCTTGTTGGGCGAAAAC
PDI1SS+10aa
Forward, GATCAGATCTCTAAAAATGAAGTTTTCTGCTG
Reverse, GATCACCGGTCCGGAGTCTTCAGGGGCCAC
Dap2SS+3aa
Forward, GATCAGATCTCTAAAAATGGAAGGTGGCGAAG
Reverse, GATCACCGGTCCGTGAGGTATACTTTTTAGCAAC

These ER targeted sfGFP-HDEL fragments were subsequently amplified using the following primers:
Forward, GCAAATGGGCGGTAGGCG
Reverse, GATCGGATCCTTACAATTCATCGTG

The resulting fragment was digested with *Bgl*II and *BamH*I and cloned into the *BamH*I site of pRS415-GPD.

The tandem dimer sfGFP (tdsfGFP) or tdTomato with KDEL ER retrieval motif was inserted at the C-terminus on the sfGFP-N1 plasmid [3, 4] was modified to contain the yeast HDEL motif using the following primers [1]:
Forward, GGACGAGCTGTACAAGGATGAATTGTAAGCG
Reverse, CGCTTACAATTCATCGTGGTACAGCTCGTCC

Kar2SS+3aa-td-sfGFP-HDEL, Pdi1+3aa-tdsfGFP-HDEL or Pdi1+3aa-tdTomato-HDEL were made by inserting Kar2SS+3aa or Pdi1SS+3aa into the *Bgl*II/*Age*I site of tdsfGFP-HDEL or tdTomato-HDEL. The Kar2SS-tdsfGFP-HDEL fragment was amplified using the following primers:
Forward, GATCACTAGTCTAAAAATGTTTTTCAACAGAC
Reverse, GATCGGATCCTTACAATTCATCGTG

The resulting fragment was digested and cloned into the *Spe*I/*BamH*I site of pRS415-GPD.
The Pdi1SS+3aa-tdsfGFP-HDEL or Pdi1SS+3aa-tdTomato-HDEL fragments were amplified using the following primers:
Forward, GCAAATGGGCGGTAGGCG
Reverse, GATCGGATCCTTACAATTCATCGTG

The resulting fragment was digested with *Bgl*II and *BamH*I and cloned into the *BamH*I site of pRS415-GPD.

The mCherry with KDEL retrieval motif inserted at the C-terminus on the mCherry-N1 plasmid was modified to contain the yeast HDEL motif using the following primers:
Forward, GGACGAGCTGTACAAGGATGAATTGTAAGCG
Reverse, CGCTTACAATTCATCGTGGTACAGCTCGTCC

Kar2SS+3aa fragment was inserted into the *Bgl*II/*Age*I site of mCherry-HDEL. The Kar2SS-mCherry-HDEL fragment was amplified using the following primers:
Forward, GATCACTAGTCTAAAAATGTTTTTCAACAGAC
Reverse, GATCGGATCCTTACAATTCATCGTG

The resulting fragment was digested and cloned into the *Spe*I/*BamH*I site of pRS415-GPD.
Kar2SS+3aa-tdsfGFP-HDEL series truncations were made by amplifying fragments using the following primers:
Forward, GCAAATGGGCGGTAGGCG,
Kar2SS+3aa-sfGFP-16aa-HDEL
Reverse, GATCGCGGCCGCTTACAATTCATCGTGCAGGATGGGCACCACC
Kar2SS+3aa-sfGFP-26aa-HDEL
Reverse, GATCGCGGCCGCTTACAATTCATCGTGGTGGCCGTTTACGTCG
Kar2SS+3aa-sfGFP-36aa-HDEL
Reverse, GATCGCGGCCGCTTACAATTCATCGTG GCCCTCGCCCTCGCCG
Kar2SS+3aa-sfGFP-46aa-HDEL
Reverse, GATCGCGGCCGCTTACAATTCATCGTGCTTCAGGGTCAGCTTG
Kar2SS+3aa-sfGFP-157aa-HDEL
Reverse, GATCGCGGCCGCTTACAATTCATCGTGCTTGTCGGCGGTG

The fragments were inserted into the *Bgl*II/*Not*I sites of sfGFP-N1plasmid and were amplified using the following primers:
Forward, GATCACTAGTCTAAAAATGTTTTCAACAGAC
Reverse, GATCGGATCCTTACAATTCATCGTG

The resulting fragments were digested and cloned into the *Spe*I/*BamH*I site of pRS415-GPD.
Kar2SS+3aa-sfGFP-HDEL series insertions were made by amplifying fragments using the following primers:
Forward, GATCACCGGTCGTGAGCAAGGGCGAG,
Kar2SS+3aa-16aa -sfGFP-HDEL
Reverse, GATCACCGGTCCCAGGATGGGCACCACC
Kar2SS+3aa-26aa -sfGFP-HDEL
Reverse, GATCACCGGTCCGTGGCCGTTTACGTCG
Kar2SS+3aa-36aa -sfGFP-HDEL
Reverse, GATCACCGGTCCGCCCTCGCCCTCGCCG

The fragments were inserted into the *Age*I site of Kar2SS-sfGFP-HDEL-N1plasmid and were amplified using the following primers:
Forward, GATCACTAGTCTAAAAATGTTTTCAACAGAC
Reverse, GATCGGATCCTTACAATTCATCGTG

The resulting fragments were digested and cloned into the *Spe*I/*BamH*I site of pRS415-GPD.
Yeast codon optimized sfGFP (yesfGFP) was purchased from GenScript (Piscataway, NJ). Kar2SS-yesfGFP-HDEL was made similarly as Kar2ss-sfGFP-HDEL.

Figure S1. Heat Shock blocks ER reflux. (A) Cells were grown to early log phase at 30°C, diluted and then grown with or without 1 μg/ml Tm at the indicated temperatures, imaged with a widefield microscope, and then images were inverted for ease of visualizing the nuclear envelope and peripheral ER. Growth at 40°C in Tm protected against significant accumulation of ER-GFP in the cytosol. Scale bar = 5 μm.

Figure S2. Tandem dimer FPs prevent stress stimulated cytosolic localization for a non-GFP FP and for the inefficient Pdi1 SS. (A) An FP construct with the poorly ER-localizing SS (Pdi1 SS-ER-sfGFP) exhibits dramatically improved ER localization at both steady state and during misfolded secretory protein accumulation (5 μg/ml Tm 2 h). (B) Maintenance of ER localization during ER stress is independent of the sfGFP sequence. The same strategy works with tdTomato [5], which has low amino acid sequence identity with sfGFP. (C) Adding the 24 a.a. $(GS)_{12}$ linker protects photoconverted ER-localized yemEos3.2 from relocating to the cytosol during Tm stress. Scale bars = 5 μm.

Supplementary Table 1. Yeast strains used in this study.

Strain Index	Description	Genotype	Reference
YFG001	Wild-type	BY4741 *Mat*α; *ura3Δ0; leu2Δ0; his3Δ1; met15Δ0*	
YFG002	Kar2-sfGFP-HDEL	Kar2-sfGFP-HDEL-HIS:: KAR2 integrated; otherwise as YFG001	[1]
YPL002	Kar2SS+3aa-sfGFP-HDEL	+Kar2SS+3aa-sfGFP-HDEL-LEU; otherwise as YFG001	[1]
YPL005	Kar2SS+10aa-sfGFP-HDEL	+Kar2SS+10aa-sfGFP-HDEL-LEU; otherwise as YFG001	This paper
YFG006	Pdi1SS+3aa-sfGFP-HDEL	+Pdi1SS+3aa-sfGFP-HDEL-LEU; otherwise as YFG001	This paper
YFG007	Pdi1SS+10aa-sfGFP-HDEL	+Pdi1SS+10aa-sfGFP-HDEL-LEU; otherwise as YFG001	This paper
YFG008	Pdi1SS+10aa-NFS-sfGFP-HDEL	+Pdi1SS+10aa-NFS-sfGFP-HDEL-LEU; otherwise as YFG001	This paper
YFG009	CpySS+3aa-sfGFP-HDEL	+CpySS+3aa-sfGFP-HDEL-LEU; otherwise as YFG001	This paper
YFG010	CpySS+10aa-sfGFP-HDEL	+CpySS+10aa-sfGFP-HDEL-LEU; otherwise as YFG001	This paper
YFG011	Scj1SS+3aa-sfGFP-HDEL	+Scj1SS+3aa-sfGFP-HDEL-LEU; otherwise as YFG001	This paper
YFG012	Scj1SS+10aa-sfGFP-HDEL	+Scj1SS+10aa-sfGFP-HDEL-LEU; otherwise as YFG001	This paper
YFG013	Dap2SS+3aa-sfGFP-HDEL	+Dap2pSS+3aa-sfGFP-HDEL-LEU; otherwise as YFG001	This paper
YFG014	Kar2+3aa-mCherry-HDEL	+Kar2+3aa-mCherry-HDEL-LEU; otherwise as YFG001	This paper
YFG015	Kar2+3aa-Td-sfGFP-HDEL	+Kar2+3aa-Td-sfGFP-HDEL-LEU; otherwise as YFG001	This paper
YFG016	Pdi1+3aa-Td-sfGFP-HDEL	+Pdi1+3aa-Td-sfGFP-HDEL-LEU; otherwise as YFG001	This paper
YFG017	Pdi1+3aa-Td-Tomato-HDEL	+Pdi1+3aa-Td-Tomato-HDEL-LEU; otherwise as YFG001	This paper
YFG018	*hrd1Δ*	*hrd1Δ::KanMX4 otherwise as YFG001*	Deletion array
YFG019	*hrd1Δ*+Kar2SS+3aa-sfGFP-HDEL	+Kar2SS+3aa-sfGFP-HDEL-LEU; otherwise as YFG022	This paper
YFG020	Kar2SS+3aa-sfGFP+16aa-HDEL	+Kar2SS+3aa-sfGFP+16aa-HDEL-LEU; otherwise as YFG001	This paper
YFG021	Kar2SS+3aa-sfGFP+26aa-HDEL	+Kar2SS+3aa-sfGFP+26aa-HDEL-LEU; otherwise as YFG001	This paper
YFG022	Kar2SS+3aa-sfGFP+36aa-HDEL	+Kar2SS+3aa-sfGFP+36aa-HDEL-LEU; otherwise as YFG001	This paper
YFG023	Kar2SS+3aa-sfGFP+46aa-HDEL	+Kar2SS+3aa-sfGFP+46aa-HDEL-LEU; otherwise as YFG001	This paper
YFG024	Kar2SS+3aa-16aa-sfGFP-HDEL	+Kar2SS+3aa-16aa-sfGFP-HDEL-LEU; otherwise as YFG001	This paper
YFG035	Kar2SS+3aa-26aa-sfGFP-HDEL	+Kar2SS+3aa-26aa-sfGFP-HDEL--LEU; otherwise as YFG001	This paper
YFG036	Kar2SS+3aa-36aa-sfGFP-HDEL	+Kar2SS+3aa-36aa-sfGFP-HDEL-LEU; otherwise as YFG001	This paper
YFG037	Pdi1-sfGFP-HDEL	Pdi1-sfGFP-HDEL-HIS:: PDI1 integrated; otherwise as YFG001	This paper
YFG038	Ero1-sfGFP	+P_{Ero1p}-Ero1-sfGFP-LEU; otherwise as YFG001	This paper
YFG039	*wt* Kar2SS+3aa-sfGFP-HDEL +UPRmcherry	+UPR-mCherry-URA +Kar2SS+3aa-sfGFP-HDEL-LEU	This paper

Supplementary References

1. Lajoie P, Moir RD, Willis IM, Snapp EL. Kar2p Availability Defines Distinct Forms of Endoplasmic Reticulum Stress in Living Cells. *Mol Biol Cell* 2012;**23**(5):955-964.
2. Lai CW, Aronson DE, Snapp EL. BiP availability distinguishes states of homeostasis and stress in the endoplasmic reticulum of living cells. *Mol Biol Cell* 2010;**21**(12):1909-1921.
3. Pedelacq JD, Cabantous S, Tran T, Terwilliger TC, Waldo GS. Engineering and characterization of a superfolder green fluorescent protein. *Nat Biotechnol* 2006;**24**(1):79-88.
4. Aronson DE, Costantini LM, Snapp EL. Superfolder GFP is fluorescent in oxidizing environments when targeted via the Sec translocon. *Traffic* 2011;**12**(5):543-548.
5. Shaner NC, Campbell RE, Steinbach PA, Giepmans BN, Palmer AE, Tsien RY. Improved monomeric red, orange and yellow fluorescent proteins derived from Discosoma sp. red fluorescent protein. *Nat Biotechnol* 2004;**22**(12):1567-1572.

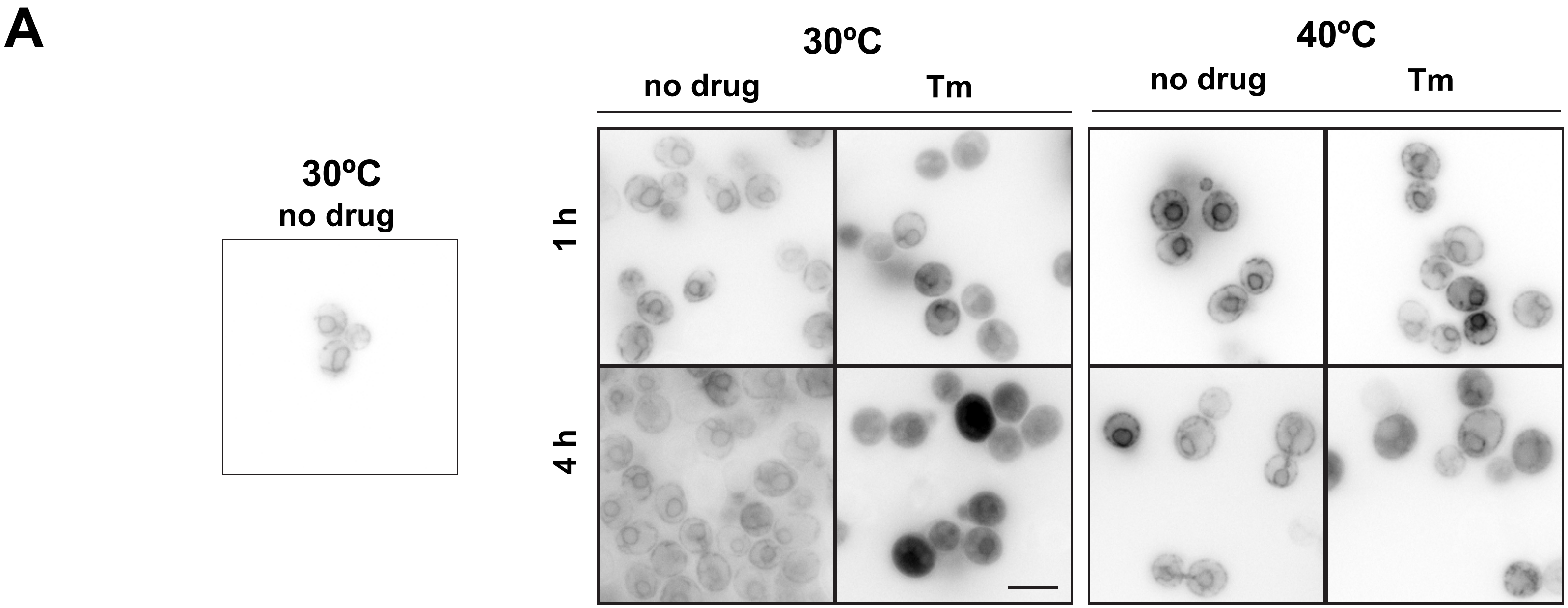

Lajoie and Snapp Supplemental Figure S1

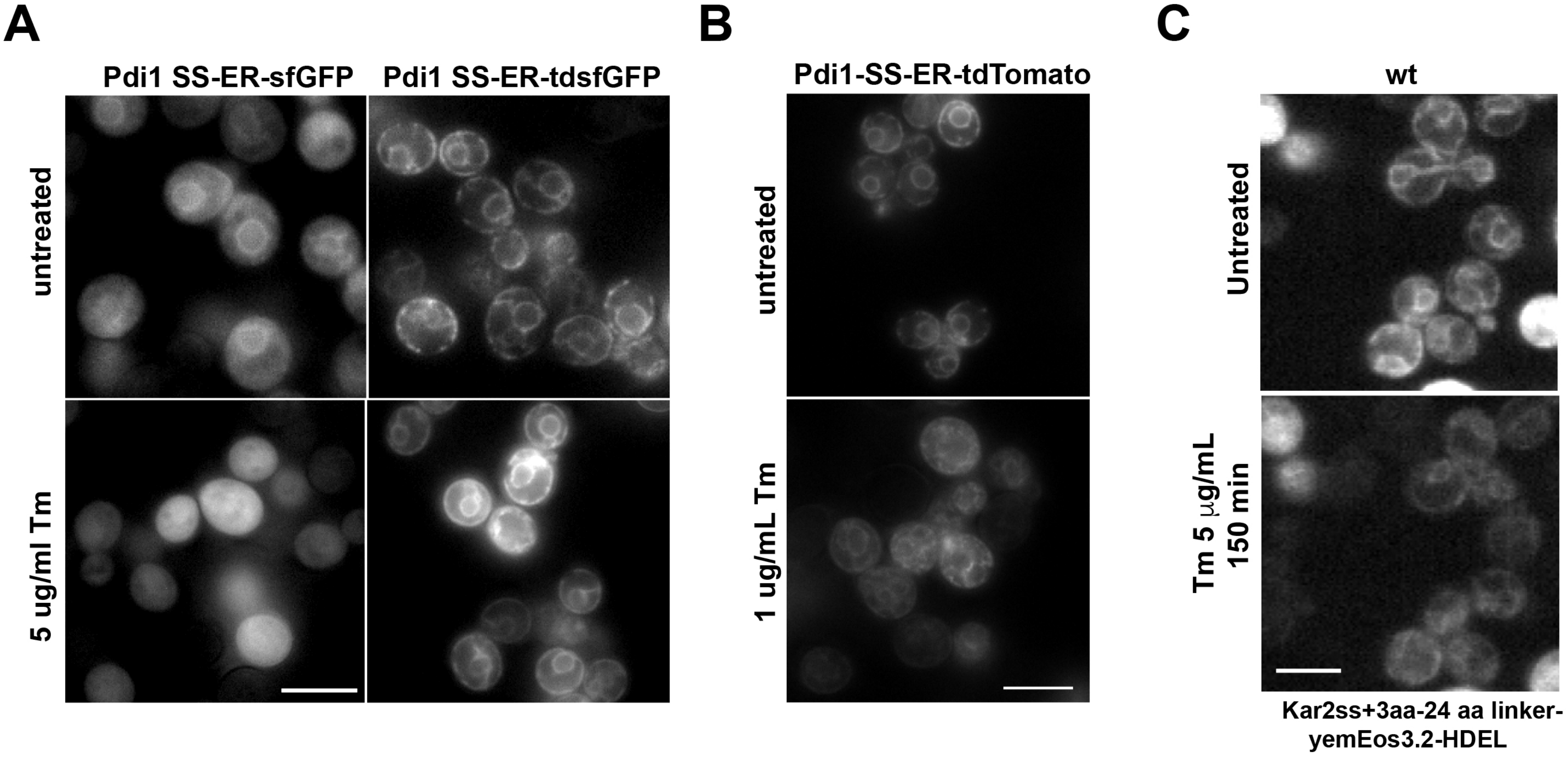

Lajoie and Snapp Supplemental Figure 2
